# Supplementary material for: Identification of photoperiod-induced specific miRNAs in the adrenal glands of Sunite sheep (Ovis aries)
Source: Front Vet Sci. 2022 Jul 22;9:888207. doi: 10.3389/fvets.2022.888207 (PMC9354845; doi:10.3389/fvets.2022.888207)
Supplement: Supplementary file 1 [file Data_Sheet_1.ZIP › Supplementary file/miRDeep2 file.zip!/miRDeep2 file/Novel_Structure/LP42A1/Novel_470.pdf]

Provisional ID	:	NC_019484.2_36054
Score total	:	56.8
Score for star read(s)	:	3.9
Score for read counts	:	49.6
Score for mfe	:	1.7
Score for randfold	:	1.6
Score for cons. seed	:	
Total read count	:	109
Mature read count	:	103
Loop read count	:	0
Star read count	:	6

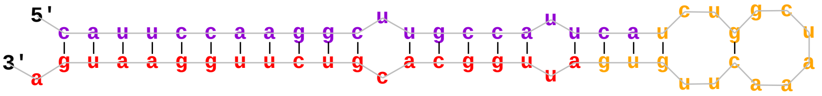

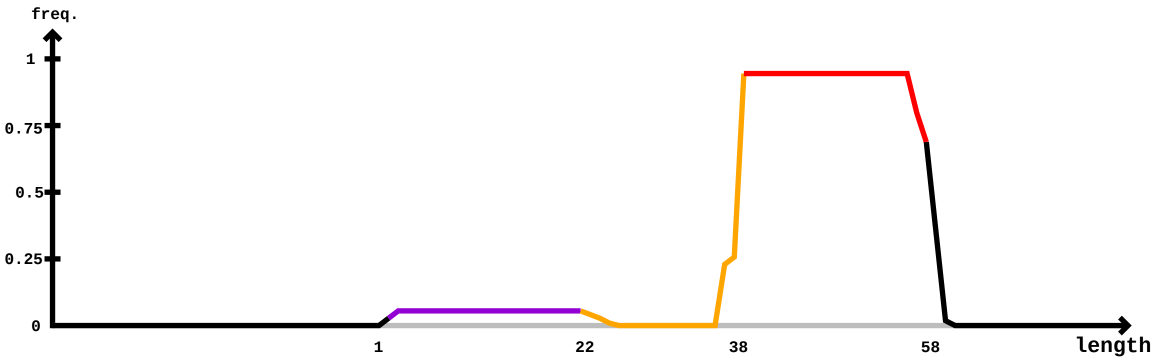

Star Mature

```
5'- uugccaccuucagcgugucugugugcuguaccccauuccaaggcuugccauucaucuggcuaaacuugugauuggcacgucuuggaaugaaguaacguacaacaaugaug   -3'    obs
    uugccaccuucagcgugucugugugcuguaccccauuccaaggcuugccauucaucuggcuaaacuugugauuggcacgucuuggaaugaaguaacguacaacaaugaug          exp
    ..((........))..((((((((((..(((..(((((((((((.(((((.((((..(......)..)))).))))).)))))))))))..)))..)))).)))..))).   reads     mm     sample
    .................................cauuccaaggcuugccauuca........................................................   3         0      seq
    ..................................auuccaaggcuugccauucauc......................................................   2         0      seq
    ..................................auuccaaggcuugccauucaucu.....................................................   1         0      seq
    ....................................................................ugauuggcacgucuuggaau......................   11        0      seq
    ....................................................................ugauuggcacgucuuggaaug.....................   4         0      seq
    ....................................................................ugauuggcacgucuuggaauga....................   5         0      seq
    ....................................................................ugauuggcacgucuuggaaugaa...................   5         0      seq
    .....................................................................gauuggcacgucuuggaaug.....................   1         0      seq
    .....................................................................gauuggcacgucuuggaauga....................   1         0      seq
    .....................................................................gauuggcacgucuuggaaugaa...................   1         0      seq
    ......................................................................auuggcacgucuuggaau......................   5         0      seq
    ......................................................................auuggcacgucuuggaaug.....................   7         0      seq
    ......................................................................auuggcacgucuuggaauga....................   42        0      seq
    ......................................................................auuggcacgucuuggaaugaa...................   19        0      seq
    ......................................................................auuggcacgucuuggaaugaag..................   2         0      seq
```
